# New Thiophene and Flavonoid from *Tagetes minuta* Leaves Growing in Saudi Arabia

**DOI:** 10.3390/molecules19032819

**Published:** 2014-03-04

**Authors:** Nawal M. Al-Musayeib, Gamal A. Mohamed, Sabrin R. M. Ibrahim, Samir A. Ross

**Affiliations:** 1 Department of Pharmacognosy, Faculty of Pharmacy, King Saud University, Riyadh 11451, Saudi Arabia; 2 Department of Pharmacognosy, Faculty of Pharmacy, Al-Azhar University, Assiut Branch, Assiut 71524, Egypt; E-Mail: gamals2001@yahoo.com; 3 Department of Pharmacognosy, Faculty of Pharmacy, Assiut University, Assiut 71526, Egypt; E-Mail: sabrinshaur@gmail.com; 4 National Center for Natural Products Research, Department of Pharmacognosy, School of Pharmacy, The University of Mississippi, University, MS 38677, USA; E-Mail: sross@olemiss.edu

**Keywords:** *Tagetes minuta*, Asteraceae, thiophene, quercetagetin, antioxidant, antimicrobial, antileishmanial, antimalarial

## Abstract

Phytochemical investigation of the methanolic extract of *Tagetes minuta* L. (Asteraceae) leaves resulted in the isolation and identification of two new compounds: 5-methyl-2,2',5',2'',5'',2''',5''',2''''-quinquethiophene (**1**) and quercetagetin-6-*O*-(6-*O*-caffeoyl-β-d-glucopyranoside) (**9**), in addition to seven known compounds: quercetin-3,6-dimethyl ether (**2**), quercetin-3-methyl ether (**3**), quercetin (**4**), axillarin-7-*O*-*β*-d-glucopyranoside (**5**), quercetagetin-3,7-dimethoxy-6-*O*-*β*-d-glucopyranoside (**6**), quercetagetin-7-methoxy-6-*O*-*β*-d-glucopyranoside (**7**), and quercetagetin-6-*O*-*β*-d-glucopyranoside (**8**). The compounds were identified by UV, IR, 1D, 2D NMR, and HRESIMS spectral data. They showed significant antioxidant activity, comparable with that of propyl gallate. Compounds **8** and **3** showed weak to moderate antileishmanial and antimalarial activities, with IC_50_ values of 31.0 μg/mL and 4.37 μg/mL, respectively.

## 1. Introduction

**Figure 1:**
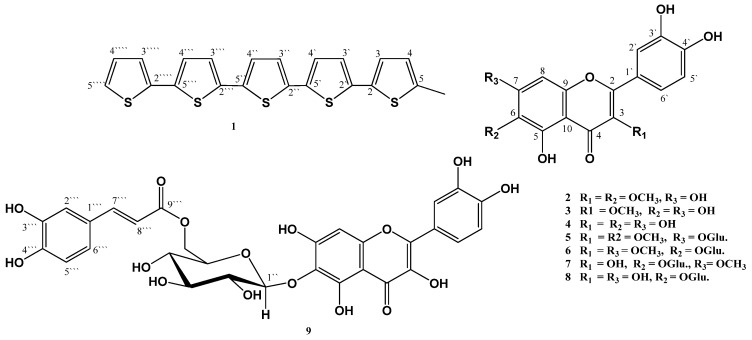
Chemical structures of the isolated compounds **1**–**9**.

The genus *Tagetes* (Asteraceae) is mainly native to the central and southern part of America. It consists of approximately 30 species [1]. Members of the genus *Tagetes* have a long history of human use as beverages, condiments, ornamentals, and medicinal decoctions. *Tagetes minuta* L. has been used as anthelmintic, diuretic, antispasmodic, and for treatment of stomach and intestinal diseases [2]. *Tagetes* oil is used as a flavor component in food products including cola and alcoholic beverages, frozen dairy desserts, candy, baked goods, gelatins, puddings, condiments, and relishes [3]. The oil has antibacterial [4], larvicidal [5], and insecticidal [6] activities. Previous phytochemical studies of *T*. *minuta* L. led to the isolation of terpenes [2,7], flavonoids [8], thiophenes, and aromatic compounds [9]. This article reports the isolation and characterization of two new compounds: 5-methyl-2,2',5',2'',5'',2''',5''',2''''-quinquethiophene (**1**) and quercetagetin-6-*O*-(6-*O*-caffeoyl-β-d-gluco-pyranoside) (**9**), together with seven known flavonoids (Figure 1). 

## 2. Results and Discussion

**Figure 2:**
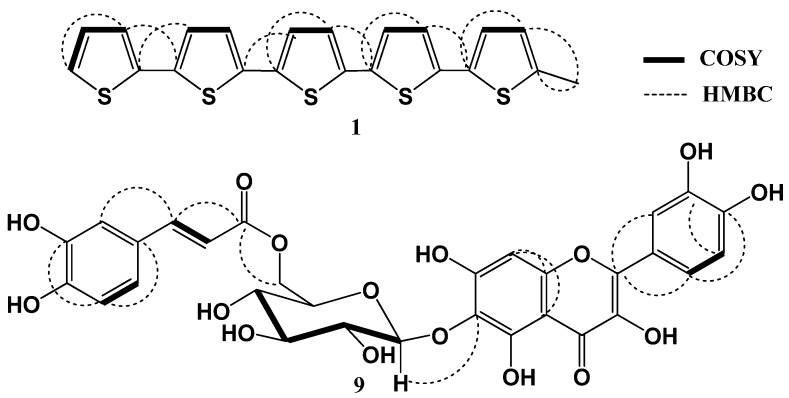
^1^H-^1^H COSY and HMBC correlations of **1** and **9**.

Compound **1** was isolated as brown needles. HRESIMS gave an [M+H]^+^ at *m*/*z* 427.6611 and 428.6613 [M+2H]^+^, which is consistent with the molecular formula C_21_H_14_S_5_, implying fifteen degrees of unsaturation. The UV absorption maxima at 387 and 334 nm indicated the presence of quinquethiophene moiety [10,11]. The ^1^H-NMR spectrum showed eleven protons signals at δ_H_ 6.64–7.21 with coupling constants 5.5–3.5 Hz characteristic for 5-subsituted quinquethiophenes [11]. Additionally, the proton signal at δ_H_ 2.42 (3H, s) indicated the presence of a methyl group (Table 1). The ^13^C-NMR spectrum exhibited twenty one carbon resonances. The multiplicities of the carbons in **1** were confirmed with DEPT and HSQC experiments, which showed one methyl, eleven methines, and nine quaternary carbons. ^1^H-^1^H COSY provided five spin systems for five thiophene rings (Figure 2). The HMBC spectrum exhibited cross peaks from methyl protons at C-5 to C-4 and C-5. In HMBC spectrum, cross-peaks from H-3 to C-2', H-3' to C-2, H-4' to C-2'', H-3'' to C-5', H-4'' to C-2''', H-3''' to C-5'', H-4''' to C-2'''', and H-3'''' to C-5'''confirmed the connectivity of thiophene rings [11]. Accordingly, **1** was 5-methyl-2, 2',5', 2'',5'',2''',5''',2''''-quinquethiophene. Compound **1** was isolated for the first time from natural origin.

**Table 1:** NMR spectral data for compound **1** (CDCl_3_, 500 and 125 MHz).

No.	δ_H_ [mult., *J* (Hz)]	δ_C_ (mult.)	HMBC
2	-	133.5 (C)	-
3	6.93 d (3.5)	125.9 (CH)	2, 4, 5
4	6.64 brs	124.0 (CH)	2, 3, 5
5	-	137.3 (C)	-
2'	-	134.2 (C)	-
3'	6.97 d (3.5)	121.2 (CH)	2
4'	6.96 d (3.5)	122.3 (CH)	2', 3', 2''
5'	-	128. 9 (C)	-
2''	-	134.7 (C)	-
3''	6.98 d (3.5)	121.5 (CH)	4''', 2'''
4''	7.02 d (3.5)	122.3 (CH)	2'''
5''	-	126.1 (C)	-
2'''	-	135.1 (C)	-
3'''	6.99 d (3.5)	125.8 (CH)	4''', 5''', 2'''
4'''	7.12 d (3.5)	121.6 (CH)	2''', 2'''', 3''', 5'''
5'''	-	132.8 (C)	-
2''''	-	135.3 (C)	-
3''''	7.04 brs	122.5 (CH)	4'''', 5'''
4''''	7.14 brd (3.5)	121.7 (CH)	2'''', 3''''
5''''	7.21 brd (5.5)	123.0 (CH)	3'''', 4''''
5-CH_3_	2.42 s	15.4 (CH_3_)	4, 5

Compound **9** was isolated as a brown residue. It gave positive tests for flavonoids [12]. The HRESIMS spectrum showed a pseudo-molecular ion peak at *m*/*z* 643.1223, consistent with a molecular formula C_30_H_26_O_16_. The UV spectrum of **9** showed absorption bands at 275 and 365 nm suggesting its flavonol nature [12]. IR spectrum showed absorption bands at 3,460 (OH), 2,976 (aromatic C-H), 1,668 (ester C=O), 1,608 (*α*,*β*-unsaturated C=O), and 1,058 (C-O) cm^−1^. Analysis of the NMR spectra of **9** showed the presence of quercetagetin, *trans*-caffeoyl, and glucopyranosyl moieties and confirmed by significant fragment ion peaks at *m*/*z* 480.0826 [M+H-caffeoyl]^+^ and *m*/*z* 318.0299 [M+H-(caffeoyl+hexose)]^+^. The ^1^H-NMR spectrum revealed the presence of six singlets signals at δ_H_ 6.59 (H-8), 8.50 (3-OH), 9.35 (3'-OH), 9.35 (4'-OH), 10.89 (7-OH), and 12.24 (5-OH). Also, it showed three coupled protons at δ_H_ 6.88 (1H, d, *J* = 7.0 Hz, H-5'), 7.57 (1H, brd, *J* = 7.0 Hz, H-6'), and 7.74 (1H, brs, H-2') for a *tri*-substituted B-ring (Table 2). Furthermore, an anomeric proton signal at δ_H_ 5.02 (1H, d, *J* = 6.5 Hz, H-1'') indicated *β*-configuration of the glycosidic linkage [13]. In addition, signals at δ_H_ 6.96 (1H, d, *J* = 1.5 Hz, H-2'''), 6.93 (1H, dd, *J* = 6.8, 1.5 Hz, H-6''' ), and 6.77 (1H, d, *J* = 6.8 Hz, H-5''') for a *tri*-substituted phenyl ring (ABX pattern) and two *trans*-coupled olefinic protons at δ_H_ 7.43 (1H, d, *J* = 16.0 Hz, H-7''') and 6.23 (1H, d, *J* = 16.0 Hz, H-8''') indicating the presence of *trans* caffeyol moiety (Table 2) [14,15,16] in which confirmed by ^13^C-NMR signals at δ_C_ 113.4 (C-8'''), 115.3 (C-5'''), 115.7 (C-2'''), 120.5 (C-6'''), 145.3 (C-4'''), 145.5 (C-7'''), 148.3 (C-3'''), and 166.5 (C-9'''). 

**Table 2:** NMR spectral data for compound **9** (DMSO-*d*_6_, 500 and 125 MHz).

No.	δ_H_ [mult., *J* (Hz)]	δ_C_ (mult.)	HMBC
2	-	148.1 (C)	-
3	-	135.6 (C)	-
4	-	176.1 (C)	-
5	-	151.5 (C)	-
6	-	129.6 (C)	-
7	-	151.4 (C)	
8	6. 59 s	93.5 (CH)	6, 7, 10
9	-	147.7 (C)	-
10	-	105.1 (C)	-
1'	-	122.0 (C)	-
2'	7.74 brs	115.5 (CH)	2, 4', 6'
3'	-	145.0 (C)	-
4'	-	147.5 (C)	-
5'	6.88 d (7.0)	115.3 (CH)	3', 6'
6'	7.57 brd (7.0)	119.9 (CH)	2, 1', 4'
1''	5.02 d (6.5)	100.9 (CH)	6
2''	3.77 dd (7.0, 9.0)	73.1 (CH)	-
3''	3.86 m	75.7 (CH)	-
4''	3.23 dd (9.0, 9.5)	69.6 (CH)	-
5''	4.35 m	77.2 (CH)	-
6''	4.41 dd (2.8, 12.0) 4.30 dd (7.0, 12.0)	64.6 (CH_2_)	9'''
1'''	-	125.2 (C)	-
2'''	6.96 d (1.5)	115.7 (CH)	6''', 7'''
3'''	-	148.3 (C)	-
4'''	-	145.3 (C)	-
5'''	6.77 d (6.8)	115.3 (CH)	3'''
6'''	6.93 dd (1.5, 6.8)	120.5 (CH)	4''', 5''', 7''', 8'''
7'''	7.43 d (16.0)	145.5 (CH)	9'''
8'''	6.23 d (16.0)	113.4 (CH)	1''', 9'''
9'''	-	166.5 (C)	-
5-OH	12.24 s	-	-
7-OH	10.89 s	-	-
3'-OH	9.35 s	-	-
4'-OH	9.35 s	-	-
3-OH	8.50 s	-	-

The ^13^C-NMR spectrum displayed fifteen carbon signals were attributed to quercetagetin skeleton [16,17] and six carbons for glucose. The multiplicity of each carbon was determined by HSQC experiment. The glucose moiety was located at C-6 based on the HMBC cross peak of H-1'' at δ_H_ 5.02 (1H, d, *J* = 6.5 Hz) to C-6 (δ_C_ 129.6) and further confirmed by its reaction with diagnostic shift reagents. In the HMBC spectrum, the methylene protons at δ_H_ 4.41 (H-6''B) and 4.30 (H-6''A) correlated with the caffeoyl carbonyl group at δ_C_ 166.5 suggesting the connectivity of caffeoyl moiety at C-6'' and confirmed by the downfield shift of C-6'' (δ_C_ 64.6). Acid hydrolysis of **9** afforded quercetagetin, caffeic acid, and *β*-d-glucose. They were identified by co-chromatography with authentic samples using (S5) [14]. Accordingly, **9** was identified as quercetagetin-6-*O*-(6-*O*-caffeoyl-β-d-glucopyranoside).

The other compounds were identified as quercetin-3,6-dimethyl ether (**2**) [18], quercetin-3-methyl ether (**3**) [18], quercetin (**4**) [18], axillarin-7-*O*-*β*-d-glucopyranoside (**5**) [19], quercetagetin-3,7-dimethoxy-6-*O*-*β*-d-glucopyranoside (**6**) [20], quercetagetin-7-methoxy-6-*O*-*β*-d-glucopyranoside (**7**) [20], and quercetagetin-6-*O*-*β*-d-glucopyranoside (**8**) [20] by comparison of their physical and spectral data with those in the literature. The antioxidant activity of the isolated compounds **2**-**9** was determined by using a DPPH free radical scavenging system. The antioxidant percentage activity ranged from 91.6 to 68.3% (Table 3). The antioxidant effect of these compounds was related to the number of free phenolic hydroxyl groups in the 3,4-dihydroxy form in their structures, which explains the close similarity of their antioxidant activity. Absence or blocking of the hydroxyl groups by a methyl or glucose moiety leads to a decrease of the antioxidant activity [21].

**Table 3:** Antioxidant activity of the isolated compounds.

Comp.	% Activity
2	81.1
3	82.4
4	91.6
5	68.3
6	69.1
7	71.3
8	83.0
9	89.1

Compounds **1**–**9** were evaluated for their antimicrobial, antimalarial and antileishmanial activities. None of the isolated compounds **1**–**9** showed any antimicrobial activity. Compound **8** showed weak antileishmanial activity with an IC_50_ 31.0 μg/mL. Compound **3** showed moderate antimalarial activity against chloroquine sensitive (D6) clones of *P*. *falciparum* with an IC_50_ of 4.37 μg/mL.

## 3. Experimental

### 3.1. General Procedures

Melting points were measured on an Electrothermal 9100 Digital Melting Point apparatus (Electrothermal Engineering Ltd, Essex, England). Optical rotation was measured with a Perkin-Elmer 241 automatic polarimeter (Perkin-Elmer Inc, Massachusetts, MA, USA). HRESIMS was recorded on a LTQ Orbitrap (ThermoFinnigan, Bremen, Germany) mass spectrometer. Low resolution mass spectra were determined using a Finnigan MAT TSQ-7000 mass spectrometer. UV spectra were recorded on a Shimadzu 1601 UV/VIS spectrophotometer (Kyoto, Japan). The IR spectra were measured on a Shimadzu Infrared-400 spectrophotometer. 1D and 2D NMR spectra were recorded on a Bruker Avance DRX 500 instrument (Bruker BioSpin, Massachusetts, MA, USA). Column chromatography separations were performed on silica gel 60 (0.04–0.063 mm), RP_18_ (0.04–0.063 mm Merck, Darmstadt, Germany), and Sephadex LH-20 (0.25–0.1 mm, Merck, Darmstadt, Germany). TLC was performed on pre-coated plates with silica gel 60 F_254_ (0.2 mm, Merck). The solvent systems used for TLC analyses were *n*-hexane/EtOAc (97:3, S1), CHCl_3_/MeOH (95:5, S2), CHCl_3_/MeOH (90:10, S3), CHCl_3_/MeOH (85:15, S4), and *n*-BuOH/HOAc/H_2_O (4:1:5, S5). 

### 3.2. Plant Material

The leaves of *Tagetes minuta* L. (Asteraceae) were collected in June 2012 from Al-Baha, Saudi Arabia. The plant was identified by Dr. A. A. Fayed, Prof. of Plant Taxonomy, Faculty of Science, Assiut University, Egypt. A voucher specimen (TM-1-2012) was deposited at the herbarium of the research center for medicinal, aromatic and poisonous plants, King Saud University.

### 3.3. Extraction and Isolation

The air-dried powdered leaves (1.1 kg) were extracted with MeOH (4 × 5 L, 24 h each) at room temperature. The combined extracts were concentrated under reduced pressure to afford a dark green residue (30.8 g) which was suspended in distilled water (250 mL) then partitioned successively between *n*-hexane (3 × 500 mL), EtOAc (3 × 500 mL), and *n*-BuOH (3 × 500 mL). Each fraction was concentrated to give *n*-hexane (4.2 g), EtOAc (3.1 g), *n*-BuOH (2.6 g), and aqueous (17.8 g) fractions. The *n*-hexane fraction (4.2 g) was subjected to vacuum liquid chromatography (VLC) using a *n*-hexane-EtOAc gradient to afford four subfractions: H-1 to H-4. Subfraction H-1 (0.52 g) was chromatographed over a silica gel column (100 g × 50 × 2 cm) using *n*-hexane/EtOAc (99:1 to 90:10) to give **1** (17 mg, brown needles). The EtOAc fraction (3.1 g) was subjected to VLC using a CHCl_3_- MeOH gradient, to afford four subfractions: E-1 to E-4. Subfraction E-1 (0.69 g) was chromatographed over a silica gel column (100 g × 50 × 2 cm) using CHCl_3_-MeOH gradients to give **2** (12 mg, yellow needles) and **3** (17 mg, yellow needles). Subfraction E-2 (0.90 g) was similarly like subfraction E-1 to give **4** (9 mg, yellow needles). Silica gel column chromatography of subfraction E-3 (0.51 g) (150 g × 50 × 3 cm) using CHCl_3_-MeOH gradients yielded **5** (11 mg, yellow residue) and **6** (7 mg, yellow residue). Subfraction E-4 (0.81 g) was chromatographed over a Sephadex LH-20 column (100 g × 50 × 3 cm) using MeOH as an eluent to give two subfractions: E-4A (295 mg) and E-4B (430 mg). Subfraction E-4B was subjected to RP_18_ column chromatography (100 g × 50 × 2 cm) using a MeOH-H_2_O gradient to afford **7** (16 mg, yellow residue). The *n*-BuOH fraction (2.6 g) was subjected to Sephadex LH-20 column chromatography (100 g × 50 × 3 cm) using MeOH as an eluent to give three subfractions: B-1 (611 mg), B-2 (355 mg), and B-3 (760 mg). Separately, subfractions B-2 and B-3, each one was chromatographed over a RP_18_ column (40 g × 25 × 1 cm) using a MeOH-H_2_O gradient to give **8** (13 mg, yellow residue) and **9** (11 mg, brown residue). The other subfractions were retained for further investigation.

### 3.4. Spectral Data

*5-Methyl-2,2',5',2',5'',2''',5''',2''''-quinquethiophene* (**1**). Brown needles (17 mg), m.p. 215–216 °C. R*_f_* 0.86, silica gel 60 F_254_ (S1). UV (MeOH): λ*_max_* 334, 387 nm. IR (KBr): *ν_max_* 2870, 1600 cm^−1^. NMR data: see Table 1. HRESIMS: *m*/*z* 427.6611 (calcd for C_21_H_15_S_5_, [M+H]^+^, 427.6609); 428.6613 (calcd for C_21_H_16_S_5_, [M+2H]^+^, 428.6609).

*Quercetagetin-6-O-(6-O-caffeoyl-β-d-glucopyranoside)* (**9**). Brown residue (11 mg), R*_f_* 0.76, silica gel 60 F_254_ (S4). [α]_D_ −176 (0.5, MeOH). UV (MeOH): λ*_max_* 275, 365 nm; +NaOMe: 282, 405 nm; +AlCl_3_: 295, 410 nm; +AlCl_3_/HCl: 293 388 nm; +NaOAc: 296, 385 nm; +NaOAc/H_3_BO_3_: 280, 385 nm. IR (KBr): *ν_max_* 3460, 2976, 1668, 1608, 1565, 1058 cm^−1^. NMR data: see Table 2. HRESIMS: *m*/*z* 643.1223 (calcd for C_30_H_27_O_16_, [M+H]^+^, 643.1221).

### 3.5. Acid Hydrolysis of **9**

Compound **9** (3 mg) was refluxed in 10 mL of 1 N HCl for 4 h. The aglycone was extracted with CHCl_3_. The sugar in the aqueous layer was identified by co-paper chromatography (PC) with authentic materials using solvent system (S5) and aniline phthalate spray as detection reagent [14].

### 3.6. Antimicrobial Assay

All the isolated compounds **2**–**9** were tested for antimicrobial activity against *Candida albicans* ATCC 90028, *Candida glabrata* ATCC90030, *Candida krusei* ATCC 6258, *Asperigillus fumigates* ATCC 90906, methicillin-resistant *Staphylococcus aureus* ATCC 33591, *Cryptococcus neoformans* ATCC 90113, *Staphylococcus aureus* ATCC 2921, *Escherichia coli* ATCC 35218, *Pseudomonus aeruginosa* ATCC 27853, and *Mycobacterium intracellulare* ATCC 23068 as described previously [22,23,24]. Ciprofloxacin and amphotericin B were used as positive standards.

### 3.7. Antimalarial Assay

The isolated compounds were tested on chloroquine sensitive (D6, Sierraleon) and resistant (W2, Indo-china) strains of *Plasmodium falciparum* using previously reported method [22,25]. Artemisinin and chloroquine were included in each assay as anti-malarial drug controls. 

### 3.8. Antileishmanial Assay

The anti-leishmanial activity of the isolated metabolites was tested *in vitro* against a culture of *L. donovani* promastigotes as previously outlined [26]. Pentamidine and amphoterecin B were used as positive standards.

### 3.9. Antioxidant Activity

The antioxidant activity of the isolated compounds **2**–**9** (20 μM) in DPPH solution (4 mg was dissolved in HPLC MeOH 50 mL to obtain a concentration 80 μg/mL) was determined as previously outlined [27,28,29].

## 4. Conclusions

In conclusion, in this study nine compounds were isolated and elucidated from *T. minuta* L. two of them (compounds **1** and **9**) are new. The antioxidant, antimicrobial, antimalarial, and antileishmanial activities of the isolated compounds were evaluated. They showed antioxidant activity ranging from 91.6% to 68.3%. Compound **8** showed weak antileishmanial activity with an IC_50_ 31.0 μg/mL, while compound **3** showed moderate antimalarial activity against chloroquine sensitive (D6) clones of *P*. *falciparum* with an IC_50_ 4.37 μg/mL.
